# Are structured interviews truly able to detect and diagnose Bipolar II disorders in epidemiological studies? The king is still nude!

**DOI:** 10.1186/1745-0179-4-28

**Published:** 2008-11-21

**Authors:** Mauro Giovanni Carta, Maria Carolina Hardoy, Tom Fryers

**Affiliations:** 1 Centro per la Terapia e la Ricerca in Salute Mentale, Dipartimento di Sanità Pubblica, Università di Cagliari, Cagliari, Italy; 2 Department of Psychiatry, University of Leicester, UK

## Abstract

**Introduction:**

A research commentary published in 2005 pointed out that the apparently low prevalence of Bipolar Disorder diagnosis as reported by epidemiological studies may be related to the under-estimate of bipolar disorder cases generally yielded by methodological instruments that are applied in such investigations.

**New data apparently challenge this notion:**

More recent publications have presented new results that apparently contradict the issues raised by the commentary, stating that the CIDI interview, which is used in the most important epidemiological studies is not only valid but highly reliable in identifying bipolar disorders.

**Commentary:**

This paper analyzes the new data and concludes that they do not give a clear indication as to how reliably the CIDI can recognize undiagnosed bipolar disorder cases. Further research studies are needed on larger "negative" (to the CIDI) samples before the field will be persuaded that CIDI really does what it is supposed to do.

## Introduction

A research commentary published in 2005 [1] pointed out that the apparently low prevalence of Bipolar Disorder diagnosis as reported by epidemiological studies may be related to the under-estimate of bipolar disorder cases generally yielded by methodological instruments that are applied in such investigations. The paper critically addressed the issue of the clinician inferential-diagnostic process, and concluded that the logical inference-process for diagnosing manic episodes (in particular phases of hypomania) in clinical settings was different from the one performed in epidemiological studies, since, in clinical practice, multiple different sources of information are being relied on, whereas, in epidemiological surveys, potentially insufficient and unreliable information is being elicited merely from patients.

One consequence of the latter approach to psychiatric diagnosis might be that a substantial percentage of individuals that have experienced manic, or more frequently, hypomanic episodes, would be diagnostically mis-identified by non-expert interviewers. They are not able to consider additional information such as elements gathered from ad hoc questions, hints by patients, socio-cultural and biographical backgrounds, and key external informants such as other patients, family members or friends.

The most likely cause is the low level of accuracy shown by structured interviews administered by lay persons.

The epidemiological studies in debate are "case finding" surveys carried out in the community ("community surveys") aiming to identify subjects who, on clinical examination, would be classified as having a mental disorder and who are not receiving appropriate treatment, as their disorder has not yet been recognized [2]. Such studies must indicate the level of accessibility of psychiatric care and thus are extremely relevant in terms of public health. The diagnostic tools used must be: a) accurate against a 'gold standard' diagnostic tool and b) reliable. The diagnostic 'gold standard' must be the most accurate tool that can determine if a subject may be considered as having the disorder considered (in this case a bipolar disorder) in terms of being suitable for treatment. In psychiatry, the 'gold standard' is the clinical diagnosis carried out in the most reliable way according to standardized diagnostic criteria, because only clinical diagnosis can decide if a subject is suitable for treatment.

The accuracy of a case finding tool (screening tool) is indicated by 'sensitivity' [proportion of true cases (verified by clinical diagnosis) identified as "positives"]; 'specificity' [proportion of true non-cases (verfied by clinical diagnosis) identified as "negatives"]; 'predictive positive value' [probability that a case identified by the test is a 'true positive'] and 'predictive negative value' [probability that a non-case identified by the test is a 'true negative'].

Reliability may be measured in terms of K (Kappa), but this measure is criticized for conditions with low frequency (as is the case of bipolar disorders) because the agreement in negatives may bias the measure [3]. For example, if two clinicians evaluated 10 subjects and both indicated one case, but it is not the same case, the agreement in 8 negatives may weigh excessively in the calculation of K. The fact that a tool is reliable does not mean that it is accurate.

In community surveys in mental health, two different methods have been used: structured interviews (yes/no answer with questions not modifiable by the interviewer) administered by non-clinicians ("lay interviewers"); and simple self-administered screening tools, which require a second level test in which all "positives" and a proportion of "negatives" must be evaluated clinically.

The issue of unreliability of structured interviews conducted by non-specialists has been further elucidated by findings from the clinical monitoring study that was carried out along-side the ESEMED project, a European epidemiological investigation. The clinical monitoring study reported a Kappa agreement coefficient (versus a clinical interview) ranging from 0.23 in Spain to 0.49 in France with regard to the diagnostic assessment of mood disorders performed with the CIDI interview [4].

The above cited commentary paper underlined that if we consider exactly what a Kappa of 0.4 implies for a disorder with an "identified" prevalence rate of 2%, we discover that the prevalence rate may have been under-estimated approximately by 1.5-times. Therefore 67% of cases may not have been identified, and 50% of identified cases may be false positives. The work concluded that, "It is legitimate to surmise that the prevalence reported by recent (extremely costly) epidemiological surveys may be doubtful".

## New data apparently challenge this notion

A more recent publication presented new results that apparently contradict the points made in the commentary, [5] stating that the CIDI interview, which is used in the most important epidemiological studies is not only valid but highly reliable in identifying bipolar disorders. The study compares the results of CIDI administered by lay persons with the semi-structured interview SCID-IV carried out by specialists. The results, while surprising, seem irrefutable: out of 40 subjects, 10 of whom were diagnosed as Bipolar I (by CIDI), 10 Bipolar II, 10 sub-threshold BPD, and 10 with no disorder in the bipolar spectrum, the concordance is almost perfect. The well-known K coefficient is almost 1 for BP I, BP II and sub-threshold cases. The article seems to have resolved the debate, particularly since the authors included as collaborators a number of excellent, well-respected clinicians, some of whom had previously questioned CIDI applicability.

## But is the king still nude? [6]

We are not convinced of the conclusions of the article. When there are suspicions that a case-detecting tool under-estimates the frequency of an ailment, the most serious problem is that of false negatives: affected individuals who are not identified as such by the "case finding" test.

As we pointed out, in the context of bipolar disorders, the crucial diagnostic issue emerges with those individuals who have experienced a hypomanic episode without being aware of it, of the range of related morbidities and co-morbidities, and of the negative outcomes of "euphoric" or "irritable" periods that it caused.

If an instrument of "case finding" underestimates a disorder, the problem is not represented by those cases which are identified as cases, In particular, as regards bipolar disorder, it is certain that a percentage of sufferers know that they have had a manic episode, since they have received treatment, and in the end have gained partial or complete illness insight. Such cases would very likely be identified through an instrument such as CIDI. However, the fact of the matter is that not all subjects reach this level of awareness, and this group would be missed in the estimate. In any case, an underestimate might arise from unidentified individuals being somewhat different from those subjects that are identified.

Let us hypothesize that the "real" rate of bipolar disorder is not 2% as many epidemiological surveys have reported, but reaches 4%, as suggested by some surveys conducted using other methods [7]. This means that half of the cases would not be recognized (false negatives).

In order to deny such a hypothesis we are interested to know that the 2% of the cases "recognized" by the test are the only ones. Their veracity can be further checked by a clinical expert. Yet, this is not the only value: we need to ensure the accuracy of the screening and the reliability of frequency estimates reported by investigations with this instrument. The crucial issue is to deny that there may exist another 2% for that same definition, that is "different" from the 2% identified.

We can refute this hypothesis if it can be demonstrated that there are, on average, just two cases, two individuals that have had a manic episode out of every 100 tested.

Let's look at how the test sample was defined. Thirty positive cases were selected, 10 each of BP I, BP II and sub-threshold disorders, and 10 "BP negative". What would the chance of recruiting a "false negative" among these 10 persons be if the true frequency in the general population were 4%? If we accepted the hypothesis of a "true" prevalence of 4% and an "identified" prevalence of 2% (among those identified by the surveys carried out applying the CIDI) it would mean that 2% of people were false negative, and therefore the likelihood of finding at least one false negative out of the 10 "negative" subjects recruited for the validation study would be 2% of 10 individuals: P = 0.20, whereas the probability of finding no false negative would very high (P = 0.80)

If one carefully analyzes the details of the study, it can be observed that the concordance among the negatives was 1, that is, among these 10, none was affected (according to both clinician assessment with SCID and lay-interviewer evaluation with CIDI). It is likely that this was the case and no false negatives were missed. Even if the prevalence was 4% and the CIDI test was not accurate, it would be difficult to note such a fallacy in a small sample that probably would not include any false negatives.

The authors claim to have weighed the sample to give a good estimate of concordance. That is to say, from this small sample of 10 true negatives with concordance of 1, the estimate of concordance would be similar even with a much larger sample such as 98% of the population.

Nevertheless, here is where the artifact originates: should 98 subjects be tested instead of 10, the CIDI might not have been as accurate and the concordance would have been lower, but with a "representative" sample of 10, it is unlikely that any false negatives would have been encountered. Thus the weight of the sample is rather arbitrary, particularly in terms of how closely it actually represented the population which it is testing. In fact, the sample was constructed in such a way as not to recognize undiagnosed cases among the normal control population. This is paradoxical for this is exactly what the main application of the test should be.

The article is certainly interesting and important both for students and for clinical epidemiologists, but some considerations should be pointed out. In terms of recognizing known cases as such, there are no doubts that the test was successful, but the results do not give a clear indication as to how reliably the CIDI can recognize undiagnosed bipolar disorder cases. And, given the straightforward way of administration of the test compared with other methods, this is exactly what it will be used for; estimates of disease prevalence are exactly what we are interested in determining with the CIDI.

## Conclusion

Further research studies are needed on larger "negative" samples before the field will be persuaded that CIDI really does what it is supposed to do.

The use of simple self administered tools in two levels community surveys on Bipolar Spectrum disorders, as the Mood Disorder Questionnaire [8,9] or the Hypomania Checklist [10,11], must be serious considered for measure the needs for care for Bipolar Spectrum Disorders in the Community.

## Competing interests

The authors declare that they have no competing interests.

## Authors' contributions

MGC had the original idea for the study, and wrote the first draft of paper, MCH and TF revised the first draft, and added some relevants points All authors read and approved the final manuscript

## Aknowledgements

We thank Fatima Obamina for valuable suggestions
